# The Primacy of β1 Integrin Activation in the Metastatic Cascade

**DOI:** 10.1371/journal.pone.0046576

**Published:** 2012-10-03

**Authors:** Hisashi Kato, Zhongji Liao, John V. Mitsios, Huan-You Wang, Elena I. Deryugina, Judith A. Varner, James P. Quigley, Sanford J. Shattil

**Affiliations:** 1 Department of Medicine, University of California San Diego, La Jolla, California, United States of America; 2 Moores UCSD Cancer Center, University of California San Diego, La Jolla, California, United States of America; 3 Department of Pathology, Moores UCSD Cancer Center, University of California San Diego, La Jolla, California, United States of America; 4 Department of Cell Biology, The Scripps Research Institute, La Jolla, California, United States of America; King's College London, United Kingdom

## Abstract

After neoplastic cells leave the primary tumor and circulate, they may extravasate from the vasculature and colonize tissues to form metastases. β1 integrins play diverse roles in tumorigenesis and tumor progression, including extravasation. In blood cells, activation of β1 integrins can be regulated by “inside-out” signals leading to extravasation from the circulation into tissues. However, a role for inside-out β1 activation in tumor cell metastasis is uncertain. Here we show that β1 integrin activation promotes tumor metastasis and that activated β1 integrin may serve as a biomarker of metastatic human melanoma. To determine whether β1 integrin activation can influence tumor cell metastasis, the β1 integrin subunit in melanoma and breast cancer cell lines was stably knocked down with shRNA and replaced with wild-type or constitutively-active β1. When tumor cells expressing constitutively-active β1 integrins were injected intravenously into chick embryos or mice, they demonstrated increased colonization of the liver when compared to cells expressing wild-type β1 integrins. Rescue expression with mutant β1 integrins revealed that tumor cell extravasation and hepatic colonization required extracellular ligand binding to β1 as well as β1 interaction with talin, an intracellular mediator of integrin activation by the Rap1 GTPase. Furthermore, shRNA-mediated knock down of talin reduced hepatic colonization by tumor cells expressing wild-type β1, but not constitutively-active β1. Overexpression in tumor cells of the tumor suppressor, Rap1GAP, inhibited Rap1 and β1 integrin activation as well as hepatic colonization. Using an antibody that detects activated β1 integrin, we found higher levels of activated β1 integrins in human metastatic melanomas compared to primary melanomas, suggesting that activated β1 integrin may serve as a biomarker of invasive tumor cells. Altogether, these studies establish that inside-out activation of β1 integrins promotes tumor cell extravasation and colonization, suggesting diagnostic and therapeutic approaches for targeting of β1 integrin signaling in neoplasia.

## Introduction

The process of cancer metastasis involves a cascade of events beginning at the primary tumor where neoplastic cells breakdown the extracellular matrix, migrate and intravasate into the vasculature [1]–[3]. Circulating tumor cells may be escorted and modified by platelets [4] and myeloid cells [5], and the metastatic process proceeds by tumor cell extravasation through blood vessels, and by seeding and colonization of a compatible niche within a distant organ [6] or even within the primary tumor [7], [8]. Tumor cells must negotiate a veritable gauntlet of environmental influences for procession through these steps of the metastatic cascade.

One mechanism that tumor cells employ during tumor progression is regulation of adhesion receptor expression [9], [10]. For example, reciprocal expression of cadherins and integrins promotes epithelial-to-mesenchymal transition [11]. Integrin αβ heterodimers expressed by tumor cells interact with extracellular matrix ligands or cellular counter-receptors to influence cell adhesion, migration, proliferation and survival [10], [12]. Within this context, the β1 integrin subunit is almost universally expressed in tumor cells, where interactions with specific matrix ligands, such as collagen, laminin and fibronectin are dictated, in part, by the identity of the integrin α subunit partner [13]. In some human solid tumors, increased expression of certain β1 integrins, for example α2β1 [14], α3β1 [15], [16], α5β1 [17], [18], or α6β1 [19], correlates with increased metastatic potential [20]–[23], and in some cases with shortened patient survival [17], [24]–[30]. On the other hand, α2β1 may suppress the progression of certain tumors [31], [32]. The therapeutic potential of β1 integrin blockade in cancer has led to current investigations of selective β1 inhibitors in animal models [33] and early clinical trials [34].

One aspect of β1 integrin function that has received relatively little attention in the cancer field is “inside-out” activation, whereby intracellular signals rapidly regulate integrin affinity for ligands through conformational changes propagated from the integrin cytoplasmic tails and transmembrane domains to the extracellular domains [35]. Thus, whereas changes in cell surface β1 integrin expression may take many minutes when regulated by receptor cycling and hours when regulated by transcription, integrin activation can take place within seconds, theoretically placing tumor cells at a relative advantage in metastatic tumor formation. Inside-out integrin signaling has been studied primarily in blood cells where β2 [36] and β3 integrin activation [37]–[39] are required for normal leukocyte trafficking and platelet aggregation, respectively. While β1 integrins are also subject to inside-out regulation in platelets [39]–[41], the role of β1 integrin activation in non-hematopoietic cells, and solid tumor cells in particular, remains to be clarified.

Based on these considerations, the current studies were carried out to investigate whether activation of β1 integrins in human tumor cells can modulate the metastatic process. We focused on the later stages of the metastatic cascade, analyzed primary and metastatic human tumors, and employed two complementary vertebrate experimental metastasis model systems. Our results establish that activated β1 integrins are expressed in certain human tumors, and that inside-out signaling to β1 integrins can determine the success or failure of tumor cell extravasation and metastatic colonization.

## Results

### Activated β1 Integrins Promote Hepatic Colonization by Tumor Cells in Experimental Metastasis Assays

**Figure 1:**
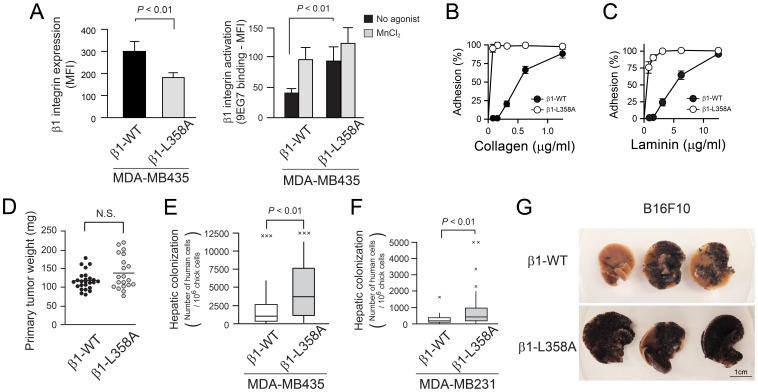
Activated β1 integrins promote hepatic colonization by tumor cells in experimental metastasis assays. (A) β1 integrin expression and activation determined by flow cytometry. Expression of β1 integrins in MDA-MB435 melanoma cells was determined by binding of antibody TS2/16. Activation of β1 integrins was determined by binding of antibody 9EG7 (basal: black bar, MnCl_2_ stimulation: gray bar). Data represent mean fluorescence intensity in arbitrary fluorescence units (MFI) ± SEM (n = 8). (B and C) Tumor cell adhesion to collagen (B) and laminin (C). β1 wild-type (WT) and β1-L358A cells were incubated in 96 well plates coated with the indicated input concentrations of collagen or laminin for 60 minutes at 37°C_._ Data are expressed as cell adhesion normalized to total input of cells. Data represent means ± SEM (n = 3). (D) Weight of primary tumors in the chick chorioallantoic membrane. 10^6^ β1-WT (n = 25) or β1-L358A MDA-MB435 cells (n = 20) were resuspended in Matrigel and inoculated on top of the membrane of 10 day-old eggs. Tumor weight was measured after seven days of incubation. (E and F) Hepatic colonization in the chick embryo experimental metastasis assay. Box plots for tumor cell colonization in the chick embryo liver five days after intravenous injection. (E) MDA-MB435 melanoma cells (β1-WT: n = 50 livers, β1-L358A: n = 32 livers) and (F) MDA-MB231 breast cancer cells (β1-WT: n = 46 livers, β1-L358A: n = 51 livers). Human tumor cells in the chick embryo liver were quantified as described in Materials and Methods. (G) Representative images of hepatic melanoma metastases in a mouse experimental metastasis system seven days after intrasplenic injection of the indicated B16F10 mouse melanoma cells. Black, melanin-containing metastatic lesions were evident macroscopically.

To begin to address a potential role for β1 integrin activation in tumor metastasis, genetically-engineered MDA-MB435 human melanoma cells were injected into the venous circulation of chick embryos and colonization to the liver was evaluated five days later. This model was employed because it is relatively rapid, enables facile quantification of human tumor cell colonization using human *Alu*-specific real-time PCR, and our preliminary experiments established that hepatic colonization in this system is dependent on β1 integrins (Fig. S1), but not β3 integrins (Fig. S2). When β1 integrin activation in MDA-MB435 cells was quantified by flow cytometry using antibody 9EG7, basal antibody binding to cells expressing constitutively-active β1-L358A was greater than the binding to cells expressing wild-type β1 (*P*<0.01) (Fig. 1A). Consistent with this, the adhesion of β1-L358A MDA-MB435 cells to low plating concentrations of collagen or laminin was increased (Fig. 1B, C), despite the fact that β1-L358A expression was approximately 50% that of wild-type β1 (Fig. 1A). There was no difference in the growth of primary tumors on the chick chorioallantoic membrane when cells expressing wild-type β1 and β1-L358A were compared (Fig. 1D). However, when MDA-MB435 tumor cells were injected intravenously into the chick embryo, the number of β1-L358A cells detected in the liver five days later was increased compared to cells expressing wild-type β1 (Fig. 1E) (*P*<0.01). This result was not confined to melanoma cells because it was also observed with genetically-engineered MDA-MB231 breast cancer cells expressing constitutively-active β1 integrin (Fig. 1F). Furthermore, the results were not confined to the chick model system because increased macroscopic hepatic metastases were observed when B16F10 mouse melanoma cells expressing β1-L358A were injected into the mouse splenic vasculature and livers examined seven days later (Fig. 1G). Thus, expression of activated β1 integrins endows circulating tumor cells with a selective advantage in hepatic colonization.

### β1 Integrins must be Competent to Bind Extracellular Ligands to Promote Hepatic Colonization by Tumor Cells

**Figure 2:**
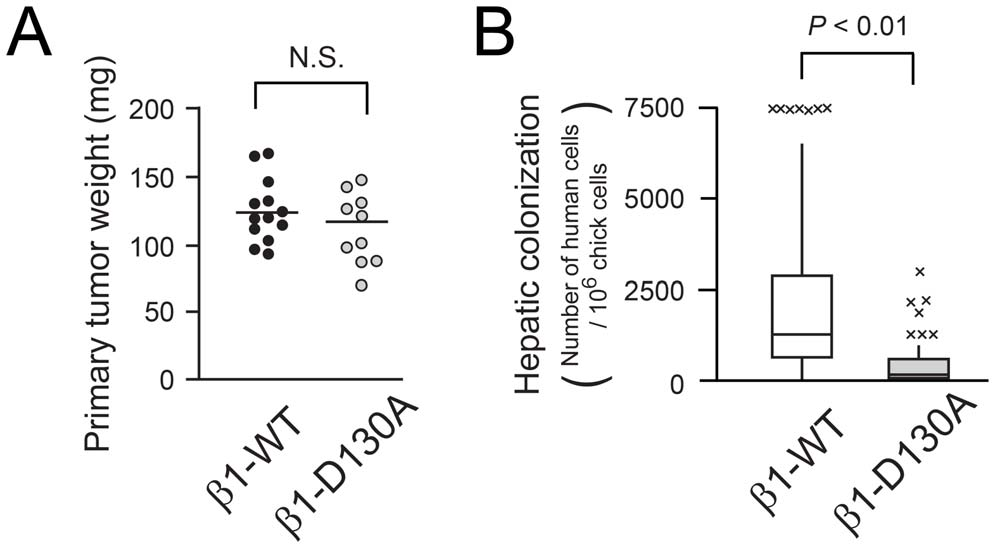
Ligand binding to β1 integrins is required for hepatic colonization by tumor cells. (A) In vivo tumor growth assay on the chick chorioallantoic membrane. Tumors growing on the membrane were weighed seven days after inoculation of 10^6^ β1-WT (n = 13) or β1-D130A MDA-MB435 cells (n = 10). (B) Box plot showing the number of β1-WT (n = 67) and β1-D130A cells (n = 57) quantified in chick embryo livers five days after intravenous tumor cell injection.

Since activated β1 integrins bind ligands with enhanced affinity, they might well be expected to affect tumor cell adhesion and motility during steps of the metastatic cascade. However, some aspects of tumor progression may be influenced by integrins in a ligand-independent manner [42]. To address whether ligand binding to β1 integrins is necessary for hepatic colonization, MDA-MB435 melanoma cells expressing wild-type β1 were compared in the chick experimental metastasis system to cells expressing equivalent levels of β1-D130A (Fig. S3A), a point mutant with impaired ligand binding [43]. As expected, the adhesion of β1-D130A melanoma cells to collagen or laminin was markedly impaired (Fig. S3B), whether or not adhesion was studied in the presence of 0.5 mM MnCl_2_ to extrinsically activate integrins (Fig. S3C). Despite this, growth of primary tumors on the chick embryo chorioallantoic membrane was not affected by the β1-D130A mutation (Fig. 2A). However, when β1-D130A cells were injected into the chick embryo venous circulation, hepatic colonization was markedly reduced compared to cells expressing wild-type β1 (Fig. 2B). Thus, activation of and ligand binding to β1 integrins are required for hepatic colonization by circulating MDA-MB435 melanoma cells but not for the growth of these cells when implanted on the chorioallantoic membrane. When specific α integrin subunits known to partner with β1 were knocked down in MDA-MB435 cells (Fig. S4A), those deficient in the α2 subunit exhibited the most profound decrease in hepatic colonization after intravenous injection into the chick embryo (Fig. S4B), suggesting that the collagen receptor, α2β1, is a major β1 integrin to promote later stages of metastasis in this system.

### Inside-out Regulation of β1 Integrin Activation Affects Hepatic Colonization by Tumor Cells

**Figure 3:**
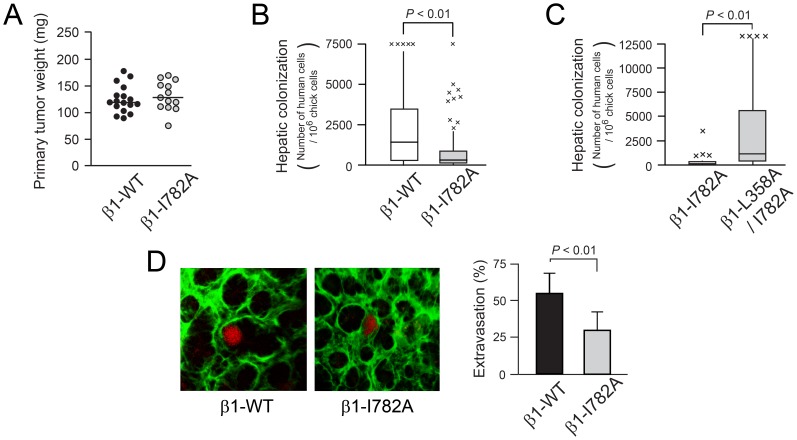
Inside-out activation of β1 integrin is required for hepatic colonization by tumor cells. (A) In vivo tumor growth assay on the chick chorioallantoic membrane. Tumors growing on the membrane were weighed seven days after inoculation of 10^6^ β1-WT (n = 18) or β1-I782A MDA-MB435 cells (n = 13). (B and C) Box plots showing the number of tumor cells colonized to the chick liver five days after intravenous injection. (B) β1-WT (n = 34) and β1-I782A cells (n = 28) and (C) β1-I782A (n = 28) and β1-L358A/I782A cells (n = 32). (D) Tumor cell extravasation from liver sinusoids. Twenty-four hours after intravenous injection of tumor cells labeled with tdTomato (red), chick embryo livers were imaged using a two-photon microscope after labeling hepatic vasculature with FITC-lectin (green). Representative images of livers from embryos injected with β1-WT or β1-I782A tumor cells depict an extravasated cell in the left panel and intravascular cell in the right panel. Three-dimensional images were digitally reconstructed and the percentage (± SEM) of extravasated cells was quantified (β1-WT: 222 cells, β1-I782A: 142 cells; six independent experiments).

Integrin activation requires interaction of the β cytoplasmic tail with talin. Therefore, to investigate a role for inside-out activation of β1 integrins in the process of hepatic colonization, a mutation (I782A) was introduced into the β1 cytoplasmic tail that disrupts talin interaction with β1 [44]. Disruption of talin binding by this mutation was confirmed in a pull-down assay using recombinant β1 tail peptides (Fig. S5A). Furthermore, MDA-MB435 cells expressing β1-I782A showed impaired adhesion to collagen and laminin in the absence of MnCl_2_ (Fig. S5B), but not in the presence of MnCl_2_ to extrinsically activate the integrin (Fig. S5C). Cells expressing β1-I782A were able to grow normally when implanted on the chick chorioallantoic membrane (Fig. 3A), but they could not promote hepatic colonization when injected intravenously (Fig. 3B). Simultaneous incorporation of the L358A activating mutation into β1-I782A restored hepatic colonization by MDA-MB435 cells (Fig. 3C), consistent with the notion that talin-dependent inside-out signaling to β1 was required for colonization and had been impaired by the I782A mutation. To specifically study the role of talin binding to β1 in tumor cell extravasation, hepatic sinusoids near the surface of the chick embryo liver were evaluated by two-photon microscopy 24 hours after intravenous injection of fluorescently-labeled tumor cells. While over 55% of cells expressing wild-type β1 had extravasated from sinusoids by this time, only ∼25% of the β1-I782A cells had done so (*P*<0.01) (Fig. 3D). Furthermore compared to tumor cells expressing wild-type β1, those expressing β1-I782A exhibited decreased total numbers in the liver 24 hours after intravenous injection (Fig. S6). Collectively, these results suggest that talin binding to β1 integrins is required for tumor cell extravasation and colonization.

**Figure 4:**
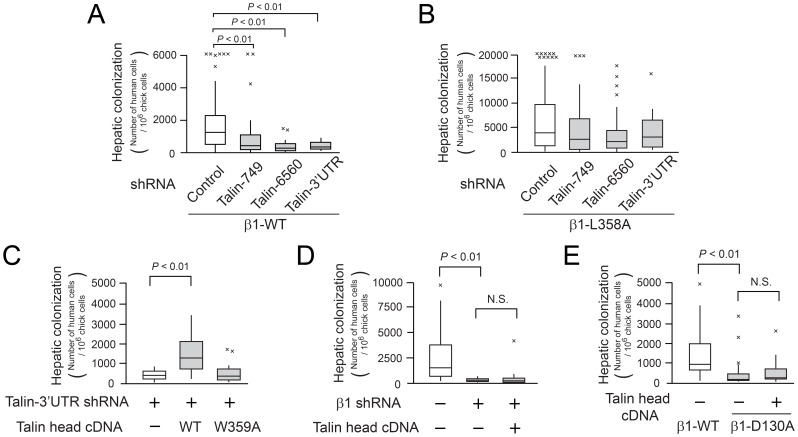
Talin-mediated β1 integrin activation is required for hepatic colonization by tumor cells. Hepatic colonization in the chick embryo experimental metastasis model. (A and B) Box plots showing the effect of talin knockdown in MDA-MB435 cells. Lentivirus encoding a control shRNA or one of three talin shRNAs were transduced into (A) MDA-MB435 β1-WT cells or (B) β1-L358A cells. Box plots depict numbers of human tumor cells quantified in the liver five days after intravenous injection into chick embryos. (C) Effect of wild-type or W359A talin head domain on hepatic colonization in talin knock down cells. (D) Talin head domain cannot rescue the blocking effect of β1 integrin knock down on hepatic colonization (control: n = 14, β1shRNA: n = 15, β1shRNA + talin head: n = 19). (E) Talin knock down cannot rescue the blocking effect of ligand binding-defective β1-D130A on hepatic colonization (β1-WT: n = 27, β1-D130A: n = 23, β1-D130A + talin head: n = 19).

To study talin directly, the protein was knocked down in MDA-MB435 with shRNA. Each of three shRNA constructs decreased talin expression (Fig. S7A), without substantially affecting β1 integrin expression (Fig. S7B). Talin knockdown decreased the adhesion of cells expressing wild-type β1 to collagen and laminin, but not the adhesion of cells expressing constitutively-active β1-L358A (Fig. S7C, D). Moreover, talin knock down decreased hepatic colonization by tumor cells expressing wild-type β1 (*P*<0.01) (Fig. 4A), but not colonization by cells expressing β1-L358A (Fig. 4B). Talin contains an N-terminal head domain and a C-terminal rod domain, and recombinant expression of the integrin-binding head domain can directly activate integrins from inside cells [45]. Indeed, overexpression of the talin head domain increased hepatic colonization by talin knock down cells expressing wild-type β1, whereas a talin head domain mutant (W359A) incapable of binding to β1 [46] failed to do so (Fig. 4C). Since the effect of the talin head domain required both the expression (Fig. 4D) and ligand binding capacity of β1 integrins (Fig. 4E), these results imply that inside-out regulation of talin binding to and activation of β1 integrins promotes hepatic colonization by tumor cells.

### A Tumor Suppressor Gene can Regulate β1 Integrin Activation and Hepatic Colonization by Tumor Cells

**Figure 5:**
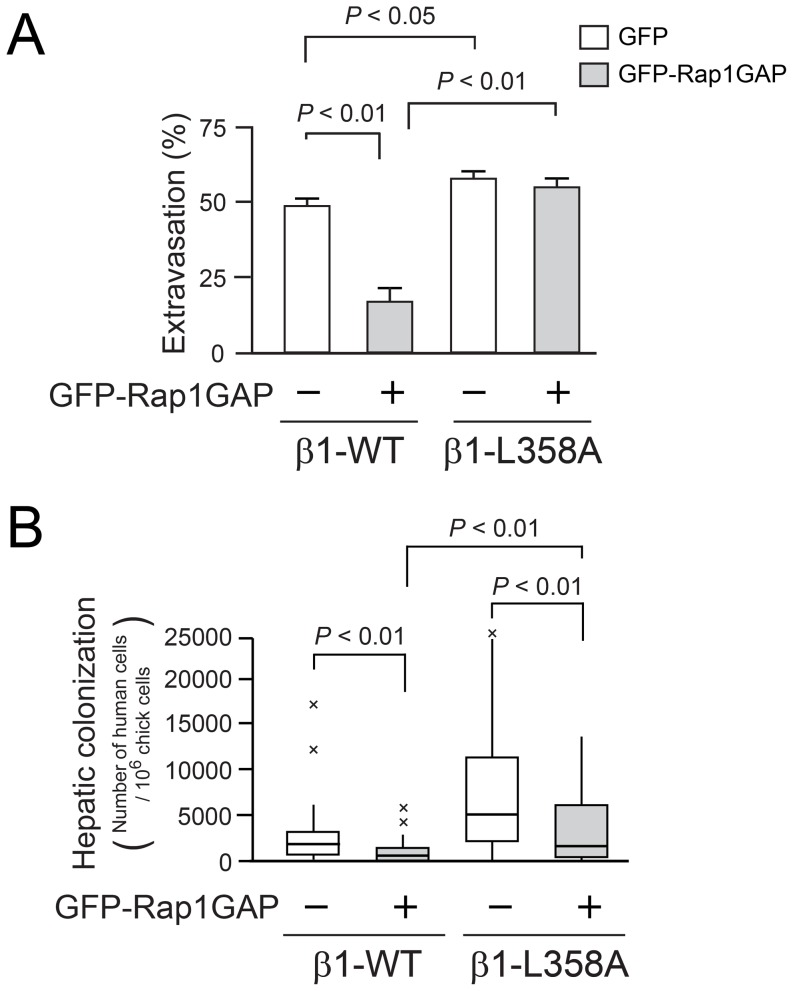
Rap1GAP suppresses hepatic colonization of tumor cells. (A) Tumor cell extravasation from liver sinusoids. Tumor cell extravasation was determined as in Figure 3 (β1-WT + GFP: 168 cells, β1-WT + GFP-Rap1GAP: 101 cells, β1-L358A + GFP: 84 cells, β1-L358A + GFP-Rap1GAP: 144 cells; five independent experiments). (B) Box plot showing the effect of Rap1GAP overexpression on hepatic colonization by MDA-MB435 β1-WT or β1-L358A cells (β1-WT + GFP: n = 28, β1-WT + GFP-Rap1GAP: n = 31, β1-L358A + GFP: n = 30, β1-L358A + GFP-Rap1GAP: n = 29).

While mutational activation of integrins in human cancer is not commonly reported, β1 integrin activation in tumor cells might be promoted by 1) stimulation of inside-out signaling through oncogenic growth factor receptor pathways, and/or 2) deletion of a tumor suppressor gene that normally functions to dampen integrin activation. One such potential tumor suppressor is Rap1GAP, which converts active Rap1-GTP to inactive Rap1-GDP, and is deleted in a number of cancers, including melanoma [47]. Since Rap1 mediates talin-dependent integrin activation [35], [48], we studied the effect of GFP-Rap1GAP on the formation of hepatic colonization. Expression of GFP-Rap1GAP in MDA-MB435 cells decreased both the levels of Rap1-GTP and cell adhesion dependent on β1 integrin (Fig. S8A, B). Moreover following intravenous injection, tumor cells expressing both wild-type β1 integrin and GFP-Rap1GAP exhibited less extravasation into the liver parenchyma (Fig. 5A) and less hepatic colonization (Fig. 5B) compared to cells expressing only wild-type β1 (*P*<0.01). However, while constitutively-active β1 integrin L358A rescued the suppressive effect of over-expressed Rap1GAP on tumor cell extravasation (Figure 5A), it was less able to rescue the suppressive effect of Rap1GAP on hepatic colonization (Figure 5B). Thus, Rap1GAP may also exert a β1 integrin activation-independent effect on tumor cell colonization after extravasation has occurred (for example on tumor cell survival, proliferation or apoptosis). Overall, these results indicate that the later stages of the metastatic cascade can be modulated by a tumor suppressor within a Rap1 signaling pathway that controls β1 integrin activation.

### Human Metastatic Tumors Express Activated β1 Integrins

**Figure 6:**
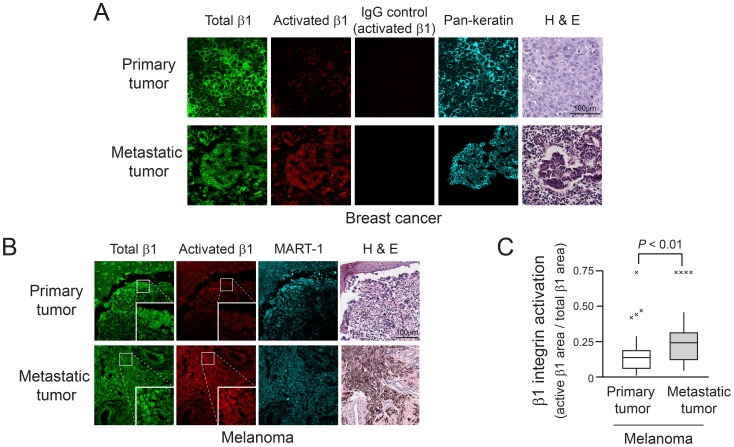
Activated β1 integrins in primary and metastatic human tumors. (A and B) Representative immunostaining of FFPE tissue sections for total β1 integrin (antibody 4B7R) and activated β1 integrin (antibody 9EG7) in primary and metastatic tumors. For detection and analysis of tumor cells, samples were stained with an antibody to pan-keratin (breast cancer) (A) or MART-1 (melanoma) (B). The breast cancer metastasis was from a lymph node and the melanoma metastasis was from the abdominal cavity. Primary and metastatic tumors were from different patients. Note that in the primary breast tumor and metastatic melanoma, activated β1 integrins were observed in some connective tissue cells as well as in tumor cells. Fluorescence images of primary and metastatic tumors were processed identically. (C) Box plot showing β1 integrin activation in primary (n = 50) and metastatic (n = 26) melanomas in a tissue microarray. The proportion of areas with activated β1 integrin to total β1 integrin within MART-1 positive melanoma cells was analyzed with Volocity software. Statistical analysis was performed by Mann-Whitney U test (Outliers, values outside of 1.5 X interquartile distance from the quartiles, are indicated by an X).

Our findings from animal models indicate a role for inside-out activation of β1 integrins in the later stages of the metastatic cascade. To begin to address a potential role for β1 integrin activation in human tumor metastasis, we assessed β1 integrin activation in formalin-fixed, paraffin-embedded (FFPE) sections of two common solid tumors, breast cancer and melanoma by using monoclonal antibody 9EG7 as a reporter for ligand-bound and activated β1 [49], [50]. The validity of using 9EG7 for this purpose was assessed in preliminary studies with genetically-engineered MDA-MB435 melanoma cells stained with both 9EG7 and antibody 4B7R (for total β1). While 9EG7 stained a relatively small sub-population of 4B7R-positive cells expressing wild-type β1, it stained the majority of cells expressing constitutively-active β1-L358A (Fig. S9). When human tumor samples were stained with 9EG7, we found a sub-population of β1 to be activated in primary and metastatic breast cancer and melanoma (Fig. 6A, B). When the tumor-bearing areas stained with antibodies 9EG7 and 4B7R were compared in a relatively large number of available melanoma samples, the proportion of activated β1 was increased in metastatic tumors compared to primary tumors (*P*<0.01) (Fig. 6C). Similarly, the fluorescence signal intensity of activated β1 relative to total β1 was increased in the metastatic tumors (median activated β1 signal intensity/total β1 intensity: 0.55 in primary tumors and 0.71 in metastatic tumors, *P*<0.01). These results suggest a role for β1 integrin activation in the metastatic cascade.

## Discussion

In the present study, we have investigated the role of inside-out signaling and activation of β1 integrins in later stages of the metastatic cascade. Given previous studies indicating the involvement of β1 integrins in multiple aspects of tumorigenesis and progression, both in animal models and in human cancers, we posited that the *activation state* of tumor cell β1 integrins, and not just the their level of expression, could impact later events in the metastatic cascade. By studying primary and metastatic human tumors and tumor cell extravasation and metastatic colonization in two vertebrate models of experimental metastasis, the following conclusions can be drawn: 1) A sub-population of β1 integrins in primary and metastatic human breast cancer and melanoma is expressed in an activated state, and in the case of melanoma, where sufficient numbers of patient samples were available for analysis, metastatic tumors expressed relatively more activated β1 integrins than primary tumors. 2) Activated β1 integrins can promote human tumor cell extravasation from the vasculature and metastatic colonization of the liver in animal models. 3) The activation state of β1 integrins in tumor cells, and subsequent extravasation and colonization, are regulated by a canonical inside-out integrin signaling pathway that includes Rap1 and talin and that requires interaction of talin with the β1 integrin cytoplasmic tail. 4) β1 integrin activation in tumor cells can be regulated by a tumor suppressor, Rap1GAP, implying that one mechanism by which this protein may affect later stages of the metastatic cascade is modulation of Rap1-dependent inside-out integrin signaling.

Increased β1 integrin expression is a prognostic factor in some human tumors [17], [24]–[30]. The present study showing relatively greater activation of β1 in human melanoma metastases compared to primary tumors suggests that future studies should investigate the activation state of β1 integrins as a potential prognostic marker in human cancers. The β1 activation-dependent antibody used in the present work detects high-affinity β1 integrins, but it also reports on ligand occupancy of the integrins and likely on integrin clustering (avidity) [50]. It is clear from our experimental metastasis studies that inside-out signaling and affinity modulation of β1 integrins can be determinants of tumor cell extravasation and colonization. However, since mechanisms of affinity and avidity regulation may differ in fine detail, future studies should also address the possible role of integrin clustering in metastasis when quantitative methods become available to differentiate β1 affinity and avidity modulation in tumor samples.

In previous mouse experiments with human breast cancer cells, the activation state of integrin αVβ3 correlated with distant metastasis [51]. In our chick embryo model system, hepatic colonization was dependent on β1 integrins, but not on αVβ3. Since tumor cell αVβ3 promotes not only tumor cell arrest in vessels but also tumor cell interaction with platelets [51], [52], the precise phases of the metastatic cascade influenced by β1 and β3 integrins may differ. For example, platelets via β3 integrins may escort and help to phenotypically reprogram circulating tumor cells [4], [53], effects not likely to be mediated by the relatively small number of β1 integrins expressed in platelets. The present studies, by focusing primarily on experimental hepatic colonization, do not address whether β1 integrin activation might also affect later stages of the metastatic cascade involving other organs or earlier stages of the cascade before cells enter the circulation from the primary tumor. However, our analysis of a human melanoma tumor array (Fig. 6C) suggests the possibility that metastases to lymph nodes and other sites may be affected by inside-out signaling to β1 integrins. While the experimental results demonstrate specific effects of β1 integrin activation on tumor cell extravasation and colonization (Fig. 3D), a limitation of the work in extrapolating to metastases in humans is that our direct intravascular injection of tumor cells in animal models does not reflect the process of cell intravasation from primary tumors. In addition, certain later processes in the metastatic cascade, such as migration within the extracellular matrix of the metastatic niche and tumor cell dormancy might be influenced by β1 integrin activation, but they were not investigated here.

The activation state of β1 integrins in tumor cells, and subsequent extravasation and metastatic colonization, were found to be regulated by an inside-out integrin signaling pathway that includes Rap1 and talin and requires interaction of talin with the β1 integrin cytoplasmic tail. Thus, the integrin activation paradigm, worked out largely with studies in hematopoietic cells [38]–[40], [54], [55], may also be relevant to circulating solid tumor cells, perhaps after they have undergone epithelial-to-mesenchymal transition. Thus, a role for β1 integrin activation may explain, in part, recent experimental work demonstrating the effects on tumor progression of manipulating the expression of Rap1, the Rap1 effector, RIAM [56], Rap1GAP [57] and talin [58]. In this regard, it is interesting to note that some members of the kindlin protein family [59]–[61], which bind integrin β tails and regulate talin-dependent integrin activation [62], [63], have also been implicated in solid tumor metastasis.

We found that β1 integrin activation in tumor cells and hepatic colonization was reduced by overexpression of the Rap1GAP tumor suppressor (Fig. 5). This suggests that one function of some tumor suppressors may be to hold integrin activation in check, an idea supported by the recent identification of a number of other putative tumor suppressors that may act at the level of integrin activation [64]–[66]. Consequently, it may be productive to move beyond β1 integrin blockade or manipulation of β1 expression as a cancer therapeutic strategy and consider the inside-out integrin activation process in tumor cells as a feature-rich set of potential therapeutic targets to limit the metastatic cascade.

## Materials and Methods

### Ethics Statement

Analysis of human breast cancer samples was approved by the UCSD Human Research Protections Program (IRB# 080911). Samples were collected as part of diagnostic or therapeutic surgery after patients gave written informed consent. No patient identifying data were available during this study. Animal experiments were conducted under a protocol approved by the University of California, San Diego Animal Subjects Committee.

### Antibodies, Cell Lines and Culture

Monoclonal antibodies to human β1 (clone TS2/16), α1, α2, α3, α5, and α6 integrin were from BioLegend (San Diego, CA). Another monoclonal antibody to human β1 integrin (clone 4B7R) was from AbD Serotec (Raleigh, NC). Monoclonal antibody to activated β1 integrin (clone 9EG7) [49] was from BD Biosciences (San Jose, CA). Polyclonal antibody to cytokeratin was from Dako (Carpinteria, CA). Monoclonal antibody to talin (clone 8D4) [45], [67] and β-actin were from Sigma Aldrich (St Louis MO). Polyclonal antibody for Rap1 was from Santa Cruz Biotechnology (Santa Cruz, CA). Monoclonal antibody for MART-1 and polyclonal antibody for human CD44 were from Spring Bioscience (Pleasonton, CA). Polyclonal antibody to cleaved caspase-3 was from Trevigen (Gaithersburg, MD). Human cancer cell lines (breast: MDA-MB231; melanoma: MDA-M435, provided by Richard Klemke, UCSD) and the mouse melanoma cell line B16F10 (provided by Mark Ginsberg, UCSD, American Tissue Culture Collection CRL-6475) were grown in DMEM supplemented with 10% fetal bovine serum, L-glutamine and antibiotics at 37°C and 5% CO_2_.

### Lentiviral Vectors for shRNA Knock Downs and Protein Expression

Lentiviral vector FG12-tdTomato and FG12-Puro were generated by substituting GFP in the original FG12 vector [68] with tdTomato [69] (kindly provided by Roger Tsien, University of California, San Diego) or the puromycin resistance gene, respectively. To express short hairpin RNA (shRNA), DNA fragments containing the human U6 promoter and shRNA amplified by PCR were cloned into FG12-tdTomato or FG12-Puro as described [70]. The following were the target sequences for human β1 integrin shRNA: AAGAGTGCCGTAACAACTGTGGTCAATCC; mouse β1 integrin shRNA: ATAAAGATCCTTTCTCAAGTCTTTT; human β3 integrin shRNA: GGCCAGATGATTCGAAGAATT; human talin-749 shRNA (talin head domain): CAATGAGCAGAAGCACAAGGCTGGCTTCC; human talin-6560 shRNA (talin rod domain): CAAGGCCGTTGCTGCTGGCAATTCCTGTC; and human talin-3′UTR shRNA: CCCAGAGTATTAACGCTCCAA; human α1 integrin shRNA: GCCATATGGAGGGAAAGGAAACAGTATGC; α2 integrin shRNA: GCTATATAGTGTGAATGAGAATGGCAATA; α3 integrin shRNA: TGGGACTTATCTGAGTATAGTTACAAGGA; α5 integrin shRNA: TCGAGACAAACTCTCGCCGATTCACATCG; α6 integrin shRNA: GACAGCTCATATTGATGTTCACTTCTTAA.

To rescue β1 integrin expression in β1 integrin knockdown tumor cells, GFP was removed from the original FG12 vector and human β1 integrin cDNA with silent mutations was cloned into FG12. To generate constitutively-active β1 integrin L358A [71], a cDNA fragment containing the L358A mutation (lower case) was amplified by PCR and cloned into the NsiI/StuI site of β1 integrin. 5′–TTGATCATTGATGCATACAATTCCgcgTCCTCAGAAGTCATTTTGG–3′ and 5′–AGCCCAGAGGCCTAATCTTGAAGCTGTCAGAATCC–3′. To produce the β1 integrin D130A [43] ligand binding-deficient mutation, a DNA fragment from the start codon to the BglII site of β1 integrin was amplified by PCR and cloned into the pCR2.1-TOPO vector and subcloned into FG12. 5′– AAAACCGGTACCCGCGGAAAAG–3′ and 5′–TTTAGATCTGTTCCAAGACTTTTTACATTCTCCAAATCGTCTTTCATTGAGTAAGACAGggcCATAAGGTAGTAGAGG–3′. To introduce the talin binding-deficient I782A [44] mutation in β1 integrin, a cDNA fragment encoding the I782A mutation was prepared by oligo annealing and cloned into the AgeI/AfeI sites of the expression vector. 5′-AATTTGAAAAGGAGAAAATGAATGCCAAATGGGACACcGGTGAAAATCCTgcaTATAAGAGC-3′, 5′-GCTCTTATAtgcAGGATTTTCACCGGTGTCCCATTTGGCATTCATTTTCTCCTTTTCA-3′. To express wild-type and mutant (W359A) [46] mouse talin head domain, cDNA 3′ to the talin F3 sub-domain was removed, and fragments encoding GFP-talin head domain were cloned into FG12 after removal of FG12 GFP segment. To generate lentiviral vector for GFP-Rap1GAP [72], Rap1GAP cDNA was cloned into pEGFP-C1 expression vector and GFP-Rap1GAP was subcloned into FG12. Lentiviruses were generated as described [68].

### Flow Cytometry

Cells were resuspended in modified Tyrode’s buffer (137 mM NaCl, 2.7 mM KCl, 3.3 mM NaH_2_PO_4_, 1.2 mM NaHCO_3_, 3.8 mM HEPES, 5.5 mM glucose, 1 mg/ml bovine serum albumin) supplemented with 1 mM MgCl_2_. To analyze total β1 integrin expression, cells were first incubated with 10 µg/ml antibody TS2/16, followed by washing and incubation with Alexa fluor 647 goat anti-mouse IgG (Invitrogen, Carlsbad, CA). Activated β1 integrin was assessed by incubating with 10 µg/ml antibody 9EG7, followed by washing and incubation with Alexa 647 goat anti-rat IgG (Invitrogen). Surface expression of β3 integrin was analyzed with monoclonal antibody SSA6 [73]. Fluorescence intensity of single, living (propidium iodide-negative) cells was determined by flow cytometry on a FACSCalibur (BD Biosciences, San Jose, CA).

### Cell Adhesion, Western Blotting and Pull-down Assays

Ninety-six well plates were coated overnight at 4°C with 100 µl of collagen type I (Sigma Aldrich, St Louis MO) or laminin-1 (Stemgent, San Diego, CA) at increasing concentrations, and then blocked with 1% BSA in phosphate-buffered saline for 1 hour at room temperature. A cell suspension in serum-free DMEM (100 µl; 10^6^ cells/ml) was applied to each well and incubated for 1 hour at 37°C in a cell culture incubator. After three washes with phosphate-buffered saline, 100 µl of substrate solution (6 mg/ml p-nitrophenyl phosphate; Sigma Aldrich, St Louis MO) in 50 mM acetic acid, pH 5.0, 1% Triton X-100 were added. After incubation for 1 hour at 37°C, the reaction was stopped with 50 µl of 1N NaOH and optical density was measured in a microplate reader at 405 nm. Cell adhesion was expressed as a percentage of total cells added to the well.

For western blotting, cells were lysed in RIPA buffer (75 mM NaCl, 1% Nonidet P-40, 1% deoxycholic acid, 0.2% sodium dodecyl sulfate, 2.5 mM MgCl_2_, 1 mM sodium orthovanadate, and proteinase inhibitor cocktail). Proteins were resolved by SDS-polyacrylamide gel electrophorsesis and transferred to nitrocellulose membranes. After blocking with 5% BSA in Tris-buffered saline, membranes were incubated with primary antibodies and then secondary antibodies conjugated to IRDye 680 or IRDye 800CW (LI-COR Biotechnology). Antibody binding was analyzed with the Odyssey imaging system (LI-COR Biotechnology).

For pull-down assays, His-tag recombinant wild-type or mutant β1 integrin cytoplasmic tail model proteins were cloned into pET15b vector, purified and conjugated to neutravidin resin as described [74]. MDA-MB435 cells were solubilized in buffer containing 1% NP-40, 150 mM NaCl, 50 mM Tris pH7.4, 1 mM sodium vanadate, 0.5 mM sodium fluoride, 1 mM leupeptin, and complete protease inhibitor cocktail (Roche Applied Science, Indianapolis, IN). After clarification, 2.0 mg of cell lysate were incubated with 100 µl of resin overnight at 4°C and bound proteins were resolved by SDS-polyacrylamide gel electrophoresis and analyzed by Western blotting. GTP-bound active Rap1 in MDA-MB435 cells was detected by pull-down assay using the Rap binding domain of RalGDS [75].

### Chick Embryo Experimental Metastasis System

Fertilized White Leghorn eggs (McIntyre poultry and fertile eggs, Lakeside CA) were incubated in a rotary incubator at 38°C with 60% humidity. The chorioallantoic membrane was dropped at day 10 of incubation. Then 10^6^ MDA-MB435 cells in 25 µl of serum-free DMEM were mixed with 25 µl of Matrigel (BD Biosciences) and inoculated onto the membrane. Following an additional 7 days of incubation, tumors growing on the membrane were excised, weighed, and prepared for histological analysis. Tumor cell colonization to the liver after intravenous injection into chick embryos was quantified by human specific *Alu*-real time PCR as described [76]. For microscopy, harvested tissues were fixed in 10% neutral-buffered formalin and embedded in paraffin. Tissue sections were prepared at 4 µm and incubated with proteinase K (20 µg/ml) for antigen retrieval. After blocking, sections were incubated with primary antibody and, after washing with phosphate-buffered saline, with the relevant fluorescein (FITC)-conjugated secondary antibody. After washing, sections were incubated with auto-fluorescence eliminator reagent (EMD Millipore, San Diego, CA) and antibody binding was analyzed in a confocal microscope (FV1000; Olympus, Center Valley, PA).

To assess tumor cell extravasation into the chick liver, 3.0×10^5^ MDA-MB435 cells labeled with tdTomato were injected into the chick embryo allantoic veins of day 12 eggs. Twenty-four hours later, 100 µl of FITC-conjugated lectin (*Lens culinaris* agglutinin, 500 µg/ml; Vector Laboratories, Burlingame, CA) were injected into the allantoic vein to label hepatic sinusoids. Livers were excised five minutes later and their surfaces were observed in a FV300 two-photon confocal microscope (Olympus, Center Valley, PA). Volocity software (PerkinElmer, Waltham, MA) was used to prepare three-dimensional images, and the number of tumor cells extravasated to the outside of the liver sinusoids was quantified.

### Analysis of Hepatic Metastasis in the Mouse

C57Bl/6 mice were anesthetized with isoflurane and the spleen was exposed by a small incision in the left flank. Then 0.75×10^6^ B16F10 mouse melanoma cells were injected into the spleen with a 30-gauge needle. Seven days later, livers were excised and imaged to assess the extent of macroscopic black tumors on the liver surface.

### Analyses of Human Tumors

A human melanoma tissue array (ME804) containing 54 cases of primary melanomas and 26 cases of metastatic melanomas was obtained from US Biomax Inc (Rockville, MD). After staining with antibodies 9EG7 for activated β1, 4B7R for total β1, and melanoma marker MART-1 or epithelial marker cytokeratin, images were captured in an FV1000 confocal microscope and NanoZoomer 2.0HT (Hamamatsu, Shizuoka, Japan). Tumor-bearing areas positive for total β1 and activated β1 integrins were analyzed using Volocity or Image J software. All tissue array images were processed and analyzed identically.

### Statistics

Mann-Whitney U test or Student’s t-test (unpaired and two-tailed) was performed as indicated.

## Supporting Information

Figure S1 **β1 integrin expression is required for hepatic colonization by tumor cells.** (A) β1 integrin expression determined by flow cytometry. The expression levels of β1 integrin in MDA-MB435 cells infected with control lentivirus or lentivirus encoding β1 integrin shRNA were determined by binding of antibody TS2/16. Data represent mean fluorescence intensity (MFI) ± SEM (n = 10). (B) Box plot showing the number of control (n = 33) and β1 knock down cells (n = 37) that colonized chick embryo livers five days after intravenous tumor cell injection.
(TIF)

Figure S2 **β3 integrin expression is not required for hepatic colonization by tumor cells.** (A) β3 integrin expression determined by flow cytometry. Expression levels of β3 integrin in MDA-MB435 cells infected with lentivirus encoding control or β3 integrin shRNA were determined by binding of antibody SSA6. Data represent mean fluorescence intensity (MFI) ± SEM (n = 4). (B) Box plot showing the number of control (n = 20) and β3 knock down cells (n = 18) that colonized chick embryo livers five days after intravenous tumor cell injection.
(TIF)

Figure S3 **Impaired adhesion of MDA-MB435 cells expressing β1-D130A.** (A) Expression levels of β1 integrins were determined by flow cytometry. (B) β1-WT or β1-D130A MDA-MB435 cells were incubated in 96 well plates coated with the indicated concentrations of collagen or laminin for 60 minutes at 37°C. (n = 3). (C) Extrinsic integrin stimulation with 0.5 mM MnCl_2_ does not induce cell adhesion of β1-D130A cells to collagen (1.25 µg/ml) or laminin (12.5 µg/ml) (n = 3). Data are expressed as cell adhesion normalized to total input of cells and represent means ± SEM.
(TIF)

Figure S4 **Collagen receptor α2β1 promotes hepatic colonization by MDA-MB435 tumor cells.** (A) Expression of the indicated α integrins before and after knock down of specific integrin α subunits was determined by flow cytometry. Data represent mean fluorescence intensity (MFI) ± SEM (α1 knock down: n = 5, α2 knock down: n = 6, α3 knock down: n = 9, α5 knock down: n = 5, α6 knock down: n = 4). (B) Box plot showing the number of MDA-MB435 cells in chick embryo livers five days after intravenous injection of tumor cells (Control: n = 92, α1 knock down: n = 52, α2 knock down: n = 52, α3 knock down: n = 42, α5 knock down: n = 38, α6 knock down: n = 35).
(TIF)

Figure S5 **Interaction of talin with β1 integrin is required for tumor cell adhesion.** (A) Pull-down of talin from MDA-MB435 cell lysates by recombinant β1-WT or β1-I782A cytoplasmic tails. Talin was detected by western blotting using anti-talin antibody 8D4. (B) β1-WT and β1-I782A MDA-MB435 cells were incubated in 96 well plates coated with increasing concentrations of collagen or laminin for 60 minutes at 37°C and cell adhesion was analyzed. (n = 3). (C) Adhesion of β1-WT and β1-I782A MDA-MB435 cells to 1.25 µg/ml collagen or 12.5 µg/ml laminin. Where indicated, integrins were activated extrinsically with 0.5 mM MnCl_2_ (n = 3± SEM).
(TIF)

Figure S6 **Hepatic colonization of tumor cells 24 hours after intravenous injection into chick embryos.** Box plot shows the number of β1-WT (n = 88) and β1-I782A cells (n = 75) present in the liver.
(TIF)

Figure S7 **Talin expression is required for β1 integrin-mediated tumor cell adhesion.** (A) Expression of talin in MDA-MB435 cells infected with lentivirus encoding control or either of three talin shRNAs was determined by Western blotting. (B) β1 integrin expression of control and talin knockdown MDA-MB435 cells was determined by flow cytometry. (C) Cell adhesion to collagen and laminin. Control and talin knockdown MDA-MB435 cells expressing β1-WT were incubated in 96 well plates coated with increasing concentrations of collagen or laminin for 60 minutes at 37°C. (n = 3). (D) Comparison of the effects of talin knock down on MDA-MB435 cells expressing either β1-WT or constitutively-active β1-L358A (n = 3± SEM).
(TIF)

Figure S8 **Rap1GAP regulates β1 integrin-mediated tumor cell adhesion.** (A) Pull-down assay for activated Rap1 in MDA-MB435 cells. Lysates from β1-WT and β1-L358A cells infected with GFP or GFP-Rap1GAP were incubated with GST-RalGDS and the binding of active GTP-Rap1 was analyzed by western blotting. MDA-MB435 cells expressing GFP-Rap1V12 and N17 served as positive and negative controls, respectively. (B) Effect of Rap1GAP overexpression on cell adhesion to collagen or laminin in cells expressing β1-WT or constitutively-active β1-L358A (n = 3± SEM).
(TIF)

Figure S9 **Activated β1 integrin in formalin-fixed paraffin-embedded tumor cell specimens.** 10^6^ β1-WT cells, β1 integrin knockdown cells (β1 shRNA) and constitutively-active β1-L358A MDA-MB435 cells were resuspended in Matrigel and implanted onto the chorioallantoic membrane of day 10 chick embryos. After seven days of additional incubation, tumors were fixed in formalin and embedded into paraffin. Tumor sections were incubated with proteinase K for antigen retrieval and stained with antibodies for total β1 integrin (4B7R) and activated β1 integrin (9EG7).
(TIF)
