# Supplementary figures and images for: The Primacy of β1 Integrin Activation in the Metastatic Cascade
Source: PLoS One. 2012 Oct 3;7(10):e46576. doi: 10.1371/journal.pone.0046576 (PMC3463578; doi:10.1371/journal.pone.0046576)

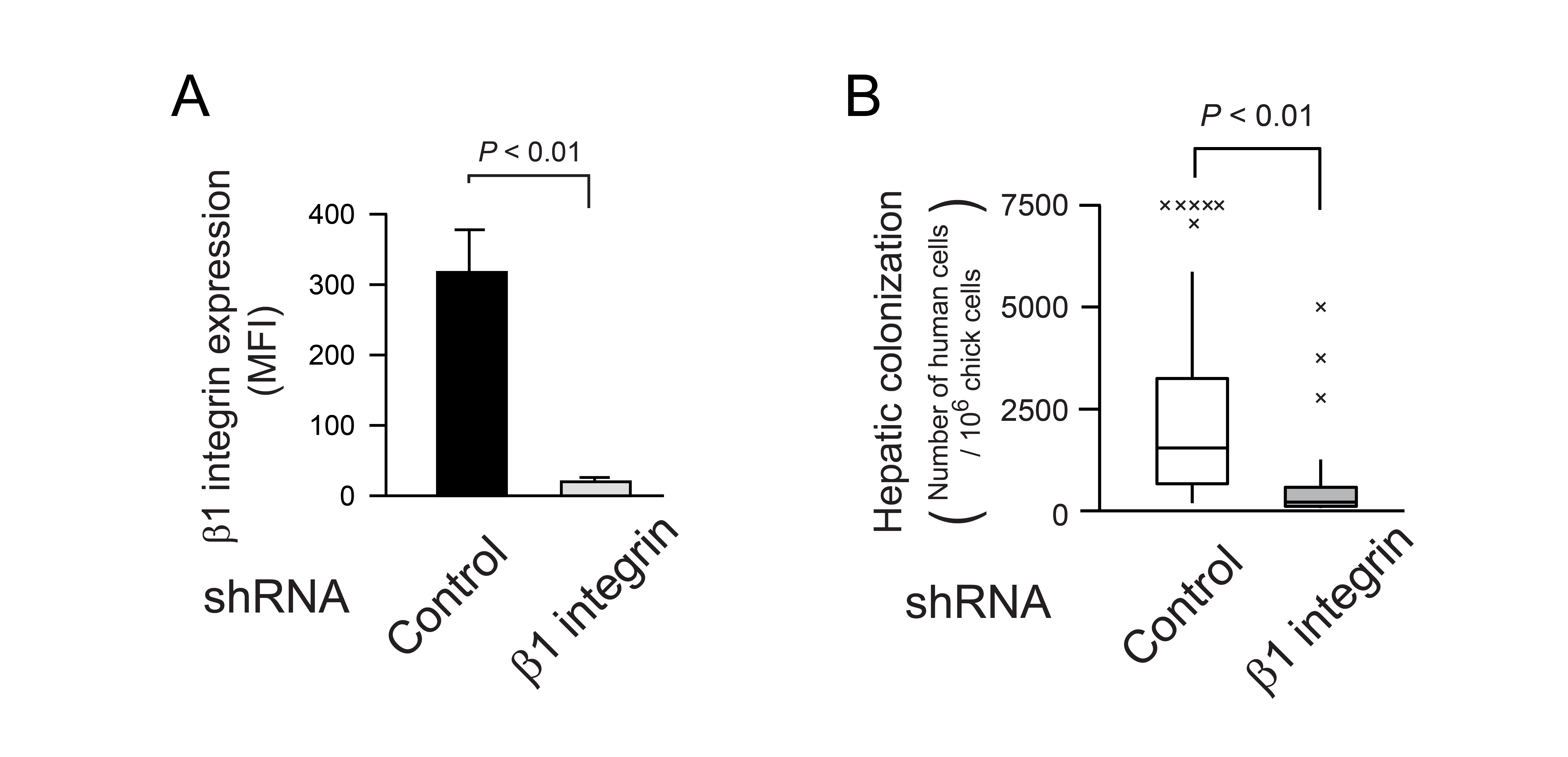

Supplement: Figure S1 — β1 integrin expression is required for hepatic colonization by tumor cells. (A) β1 integrin expression determined by flow cytometry. The expression levels of β1 integrin in MDA-MB435 cells infected with control lentivirus or lentivirus encoding β1 integrin shRNA were determined by binding of antibody TS2/16. Data represent mean fluorescence intensity (MFI) ± SEM (n = 10). (B) Box plot showing the number of control (n = 33) and β1 knock down cells (n = 37) that colonized chick embryo livers five days after intravenous tumor cell injection. (TIF) [file pone.0046576.s001.tif]

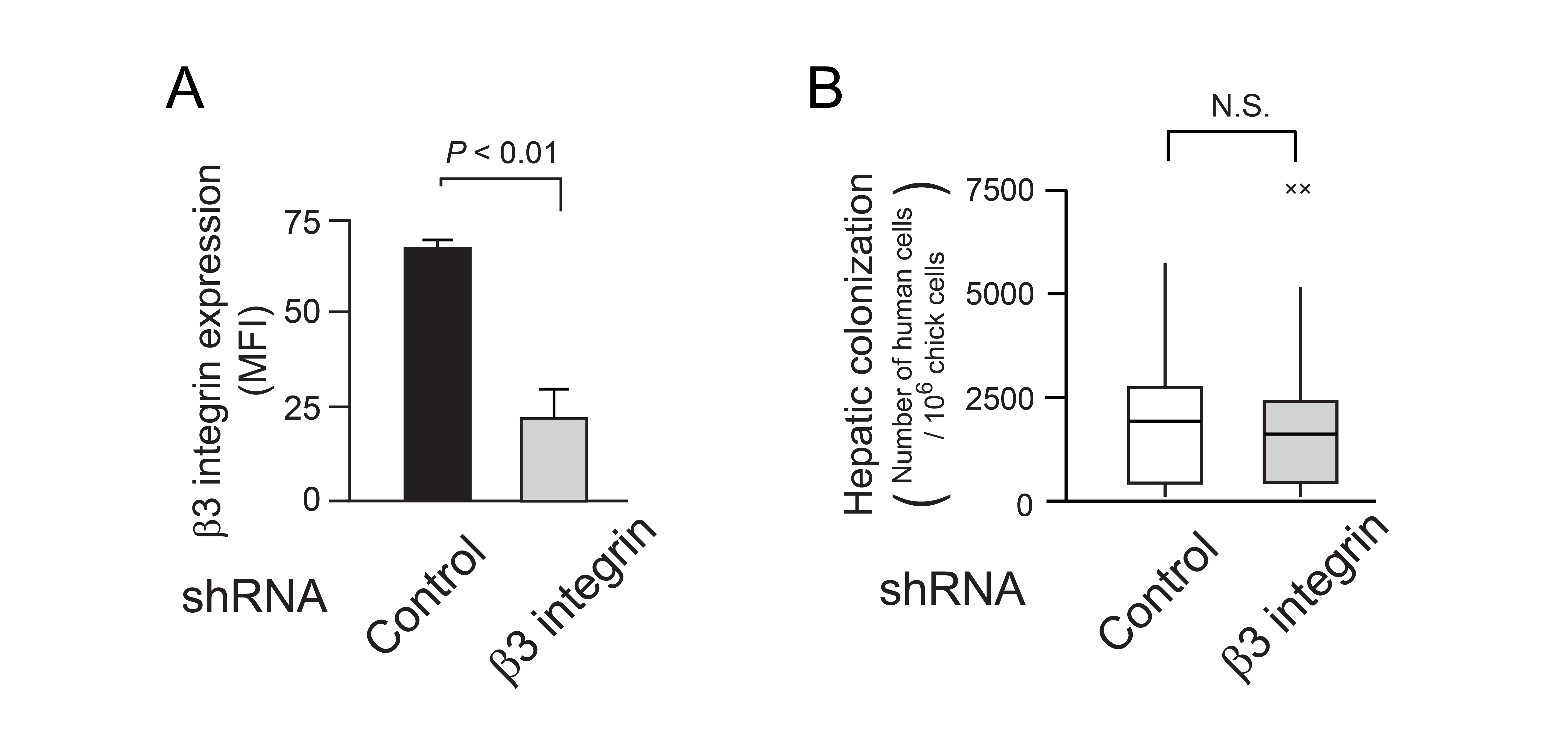

Supplement: Figure S2 — β3 integrin expression is not required for hepatic colonization by tumor cells. (A) β3 integrin expression determined by flow cytometry. Expression levels of β3 integrin in MDA-MB435 cells infected with lentivirus encoding control or β3 integrin shRNA were determined by binding of antibody SSA6. Data represent mean fluorescence intensity (MFI) ± SEM (n = 4). (B) Box plot showing the number of control (n = 20) and β3 knock down cells (n = 18) that colonized chick embryo livers five days after intravenous tumor cell injection. (TIF) [file pone.0046576.s002.tif]

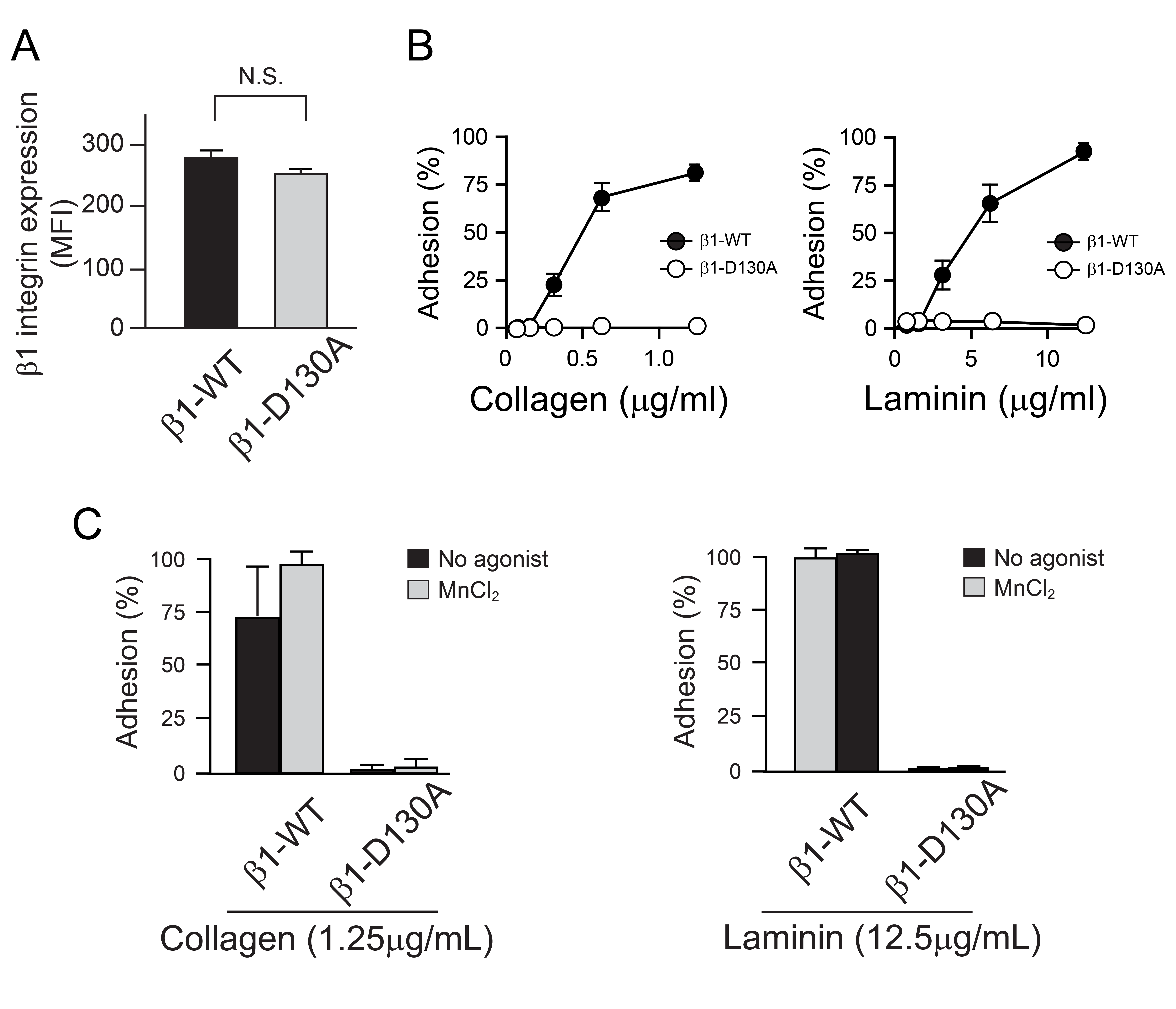

Supplement: Figure S3 — Impaired adhesion of MDA-MB435 cells expressing β1-D130A. (A) Expression levels of β1 integrins were determined by flow cytometry. (B) β1-WT or β1-D130A MDA-MB435 cells were incubated in 96 well plates coated with the indicated concentrations of collagen or laminin for 60 minutes at 37°C. (n = 3). (C) Extrinsic integrin stimulation with 0.5 mM MnCl2 does not induce cell adhesion of β1-D130A cells to collagen (1.25 µg/ml) or laminin (12.5 µg/ml) (n = 3). Data are expressed as cell adhesion normalized to total input of cells and represent means ± SEM. (TIF) [file pone.0046576.s003.tif]

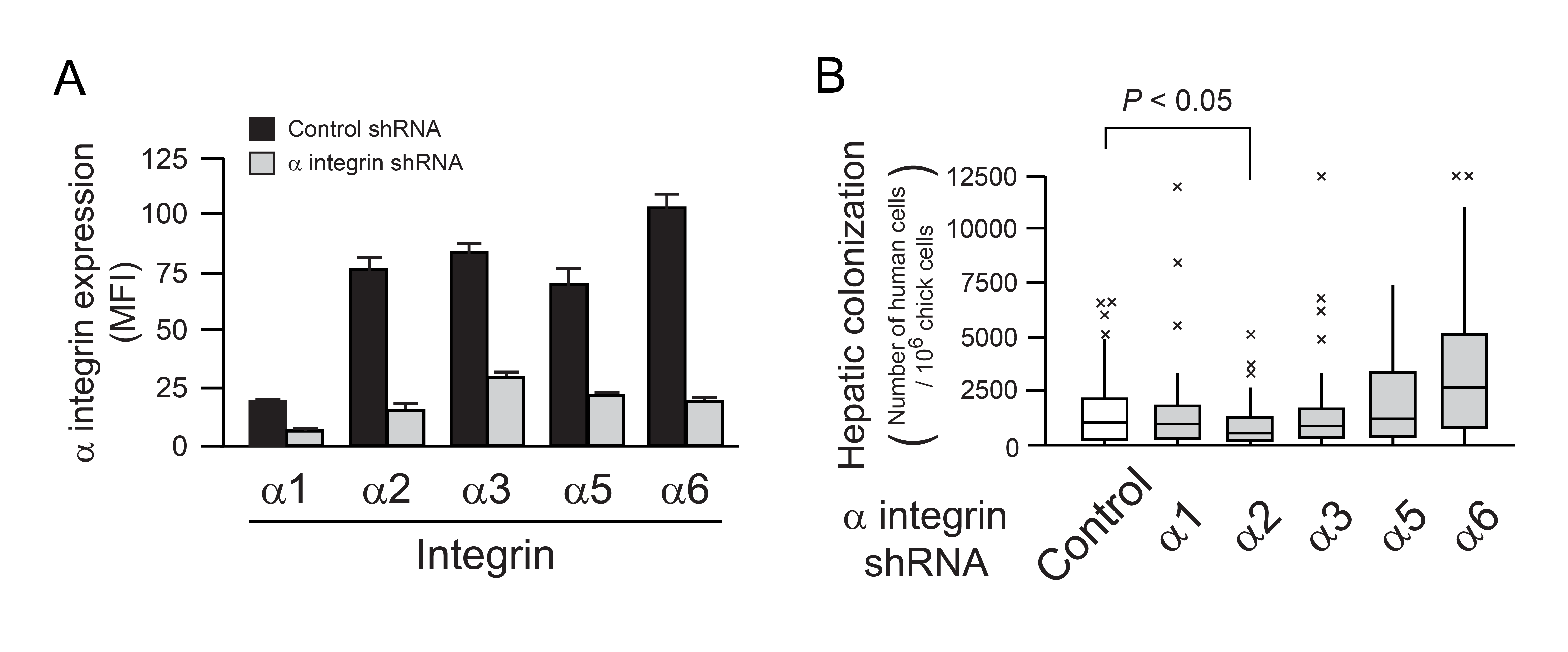

Supplement: Figure S4 — Collagen receptor α2β1 promotes hepatic colonization by MDA-MB435 tumor cells. (A) Expression of the indicated α integrins before and after knock down of specific integrin α subunits was determined by flow cytometry. Data represent mean fluorescence intensity (MFI) ± SEM (α1 knock down: n = 5, α2 knock down: n = 6, α3 knock down: n = 9, α5 knock down: n = 5, α6 knock down: n = 4). (B) Box plot showing the number of MDA-MB435 cells in chick embryo livers five days after intravenous injection of tumor cells (Control: n = 92, α1 knock down: n = 52, α2 knock down: n = 52, α3 knock down: n = 42, α5 knock down: n = 38, α6 knock down: n = 35). (TIF) [file pone.0046576.s004.tif]

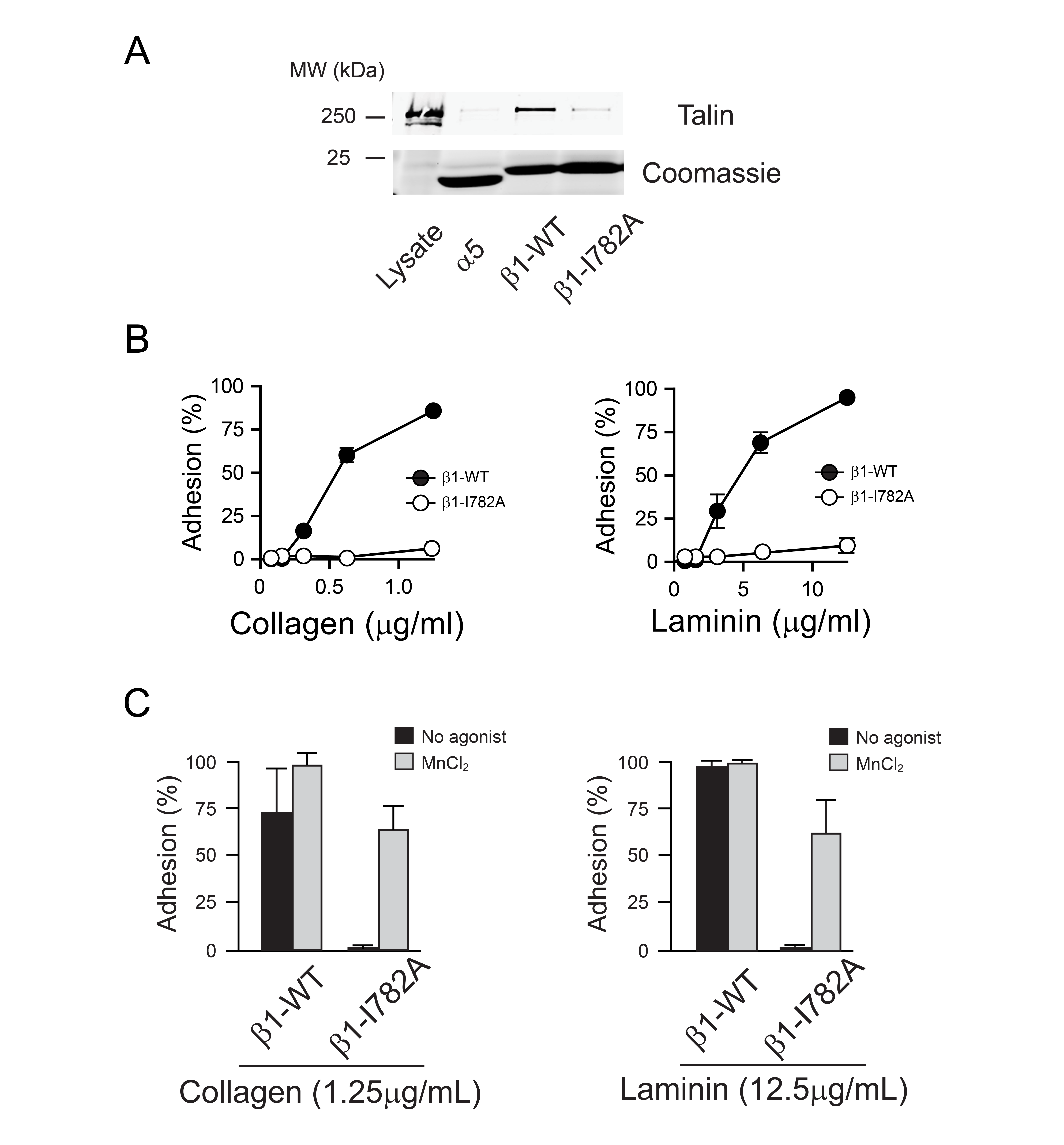

Supplement: Figure S5 — Interaction of talin with β1 integrin is required for tumor cell adhesion. (A) Pull-down of talin from MDA-MB435 cell lysates by recombinant β1-WT or β1-I782A cytoplasmic tails. Talin was detected by western blotting using anti-talin antibody 8D4. (B) β1-WT and β1-I782A MDA-MB435 cells were incubated in 96 well plates coated with increasing concentrations of collagen or laminin for 60 minutes at 37°C and cell adhesion was analyzed. (n = 3). (C) Adhesion of β1-WT and β1-I782A MDA-MB435 cells to 1.25 µg/ml collagen or 12.5 µg/ml laminin. Where indicated, integrins were activated extrinsically with 0.5 mM MnCl2 (n = 3± SEM). (TIF) [file pone.0046576.s005.tif]

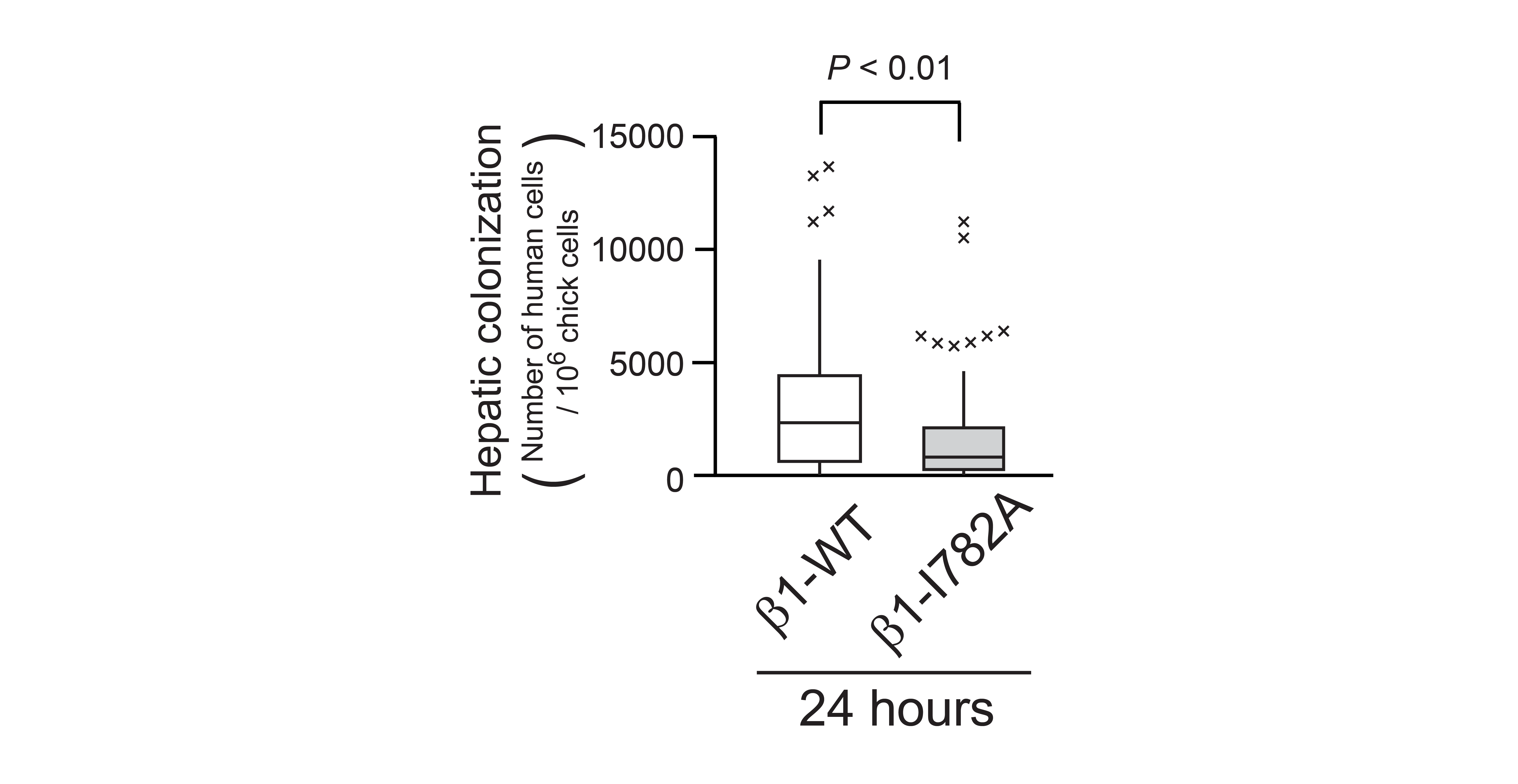

Supplement: Figure S6 — Hepatic colonization of tumor cells 24 hours after intravenous injection into chick embryos. Box plot shows the number of β1-WT (n = 88) and β1-I782A cells (n = 75) present in the liver. (TIF) [file pone.0046576.s006.tif]

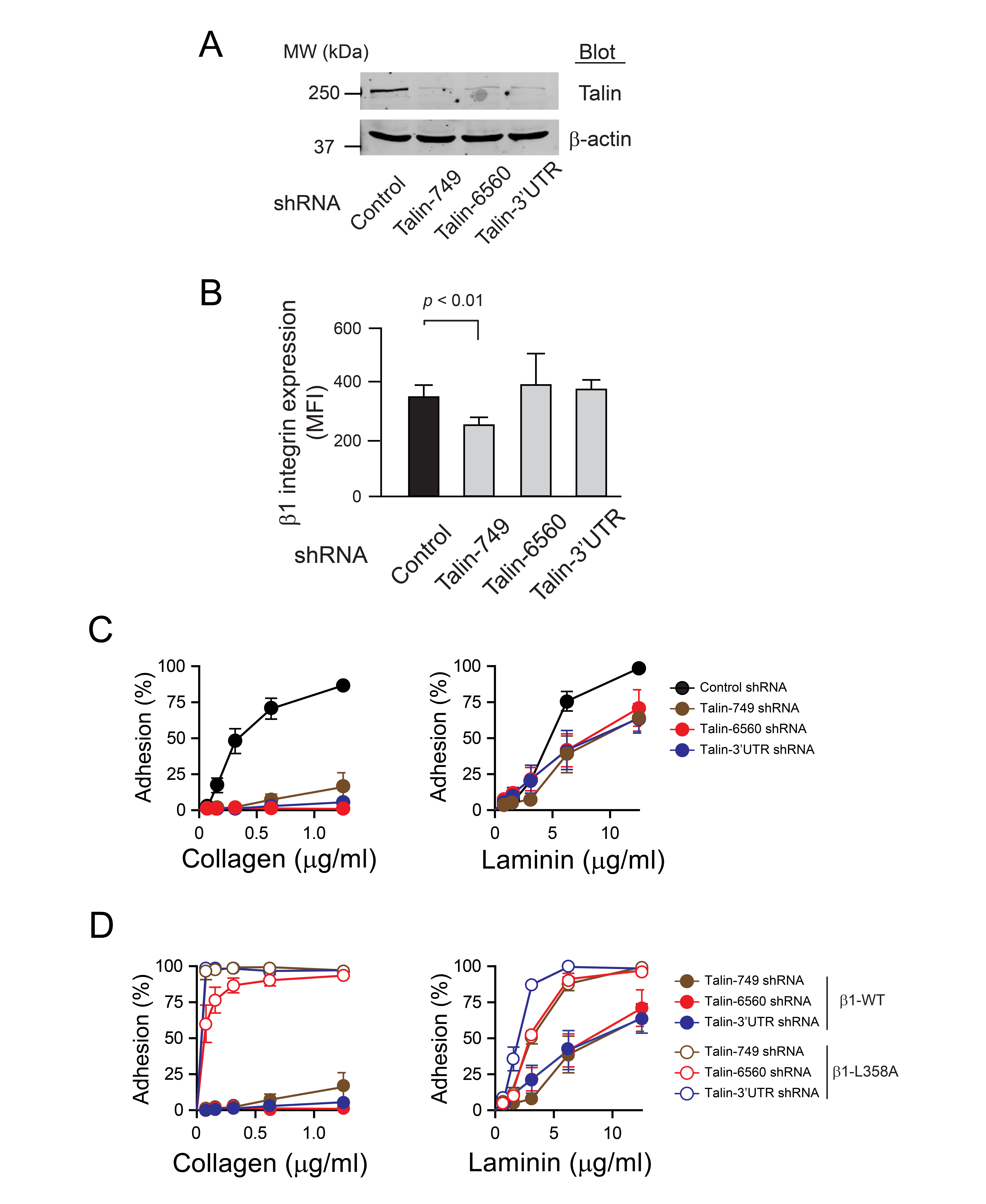

Supplement: Figure S7 — Talin expression is required for β1 integrin-mediated tumor cell adhesion. (A) Expression of talin in MDA-MB435 cells infected with lentivirus encoding control or either of three talin shRNAs was determined by Western blotting. (B) β1 integrin expression of control and talin knockdown MDA-MB435 cells was determined by flow cytometry. (C) Cell adhesion to collagen and laminin. Control and talin knockdown MDA-MB435 cells expressing β1-WT were incubated in 96 well plates coated with increasing concentrations of collagen or laminin for 60 minutes at 37°C. (n = 3). (D) Comparison of the effects of talin knock down on MDA-MB435 cells expressing either β1-WT or constitutively-active β1-L358A (n = 3± SEM). (TIF) [file pone.0046576.s007.tif]

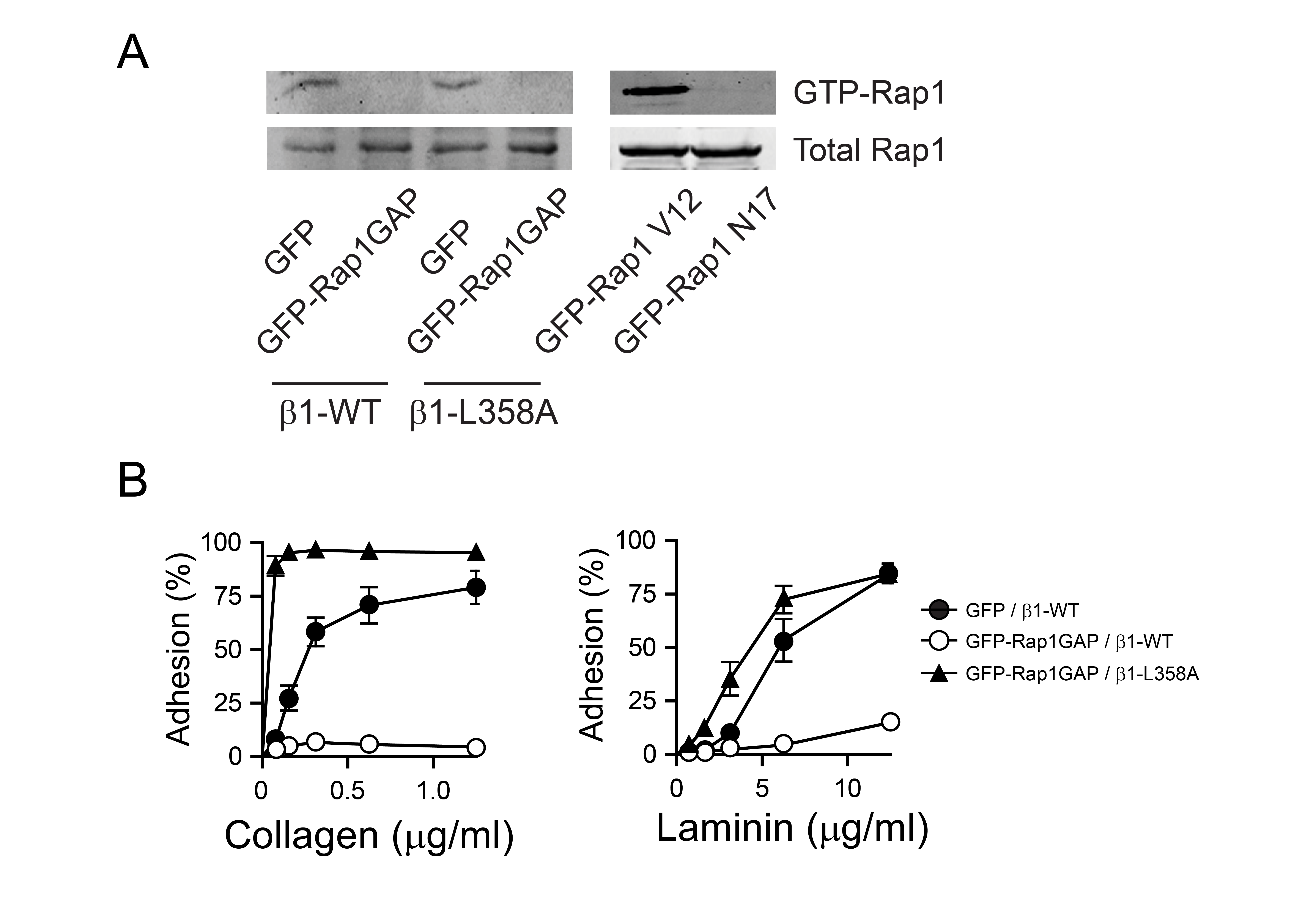

Supplement: Figure S8 — Rap1GAP regulates β1 integrin-mediated tumor cell adhesion. (A) Pull-down assay for activated Rap1 in MDA-MB435 cells. Lysates from β1-WT and β1-L358A cells infected with GFP or GFP-Rap1GAP were incubated with GST-RalGDS and the binding of active GTP-Rap1 was analyzed by western blotting. MDA-MB435 cells expressing GFP-Rap1V12 and N17 served as positive and negative controls, respectively. (B) Effect of Rap1GAP overexpression on cell adhesion to collagen or laminin in cells expressing β1-WT or constitutively-active β1-L358A (n = 3± SEM). (TIF) [file pone.0046576.s008.tif]

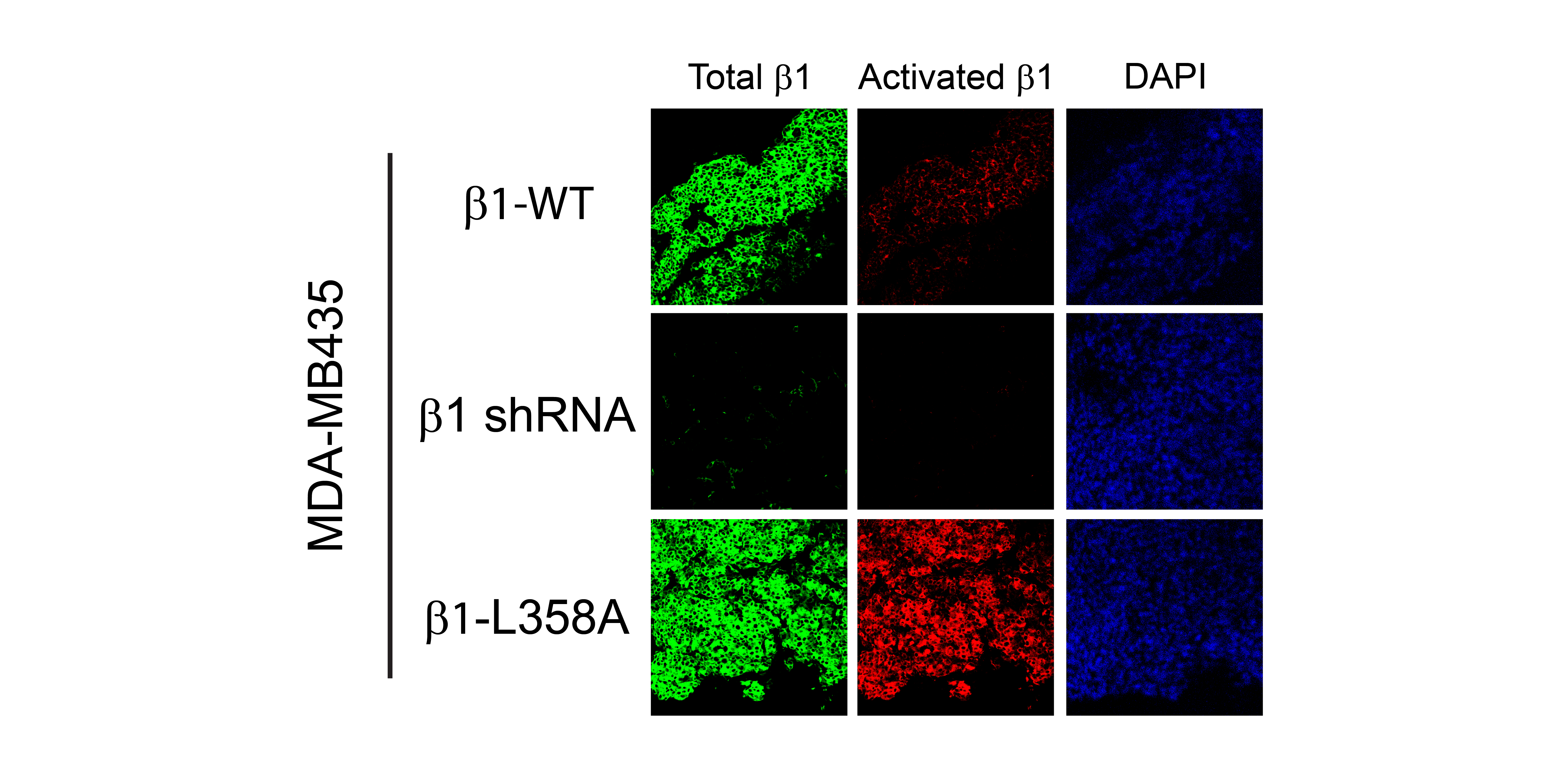

Supplement: Figure S9 — Activated β1 integrin in formalin-fixed paraffin-embedded tumor cell specimens. 106 β1-WT cells, β1 integrin knockdown cells (β1 shRNA) and constitutively-active β1-L358A MDA-MB435 cells were resuspended in Matrigel and implanted onto the chorioallantoic membrane of day 10 chick embryos. After seven days of additional incubation, tumors were fixed in formalin and embedded into paraffin. Tumor sections were incubated with proteinase K for antigen retrieval and stained with antibodies for total β1 integrin (4B7R) and activated β1 integrin (9EG7). (TIF) [file pone.0046576.s009.tif]
